# Supplementary figures and images for: Porphyromonas gingivalis lipopolysaccharide promotes T-hel per17 cell differentiation by upregulating Delta-like ligand 4 expression on CD14+ monocytes
Source: PeerJ. 2021 Apr 23;9:e11094. doi: 10.7717/peerj.11094 (PMC8074840; doi:10.7717/peerj.11094)

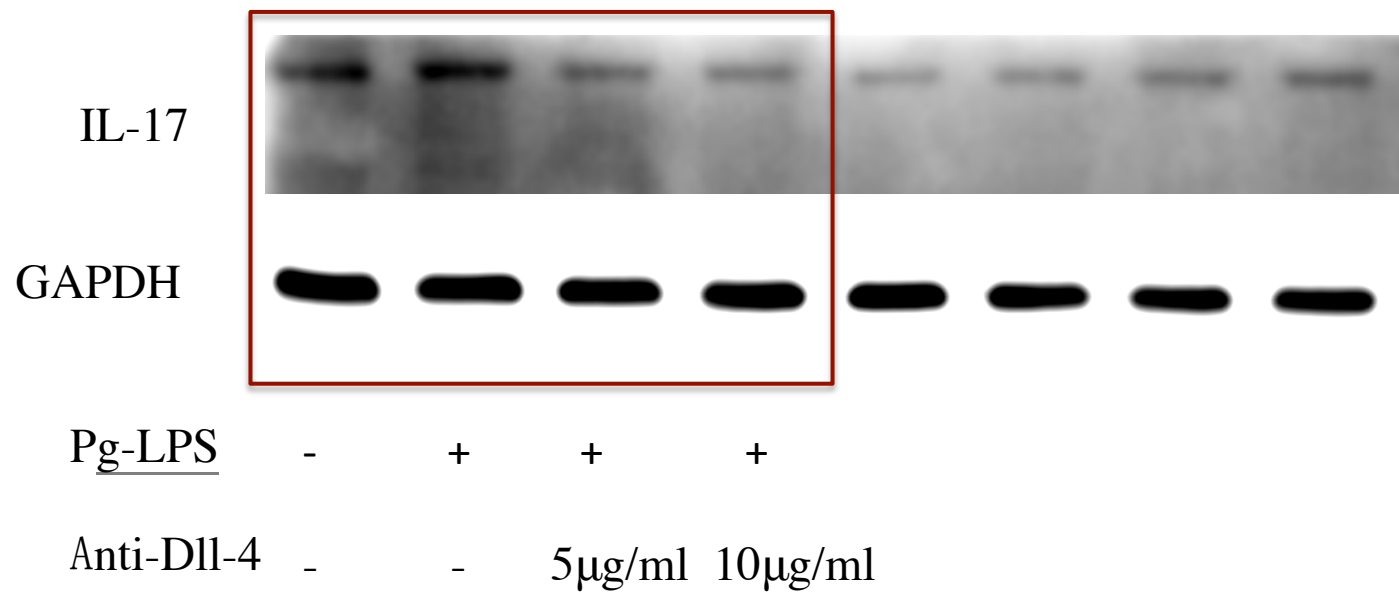

Supplement: Supplemental Information 3 — GAPDH was used as the protein loading control. [file peerj-09-11094-s003.pdf]
